# Evolutionary Analysis of a Parrot Bornavirus 2 Detected in a Sulphur-Crested Cockatoo (*Cacatua galerita*) Suggests a South American Ancestor

**DOI:** 10.3390/ani14010047

**Published:** 2023-12-22

**Authors:** Ruy D. Chacón, Christian J. Sánchez-Llatas, Andrea J. Diaz Forero, Marta B. Guimarães, Sarah L. Pajuelo, Claudete S. Astolfi-Ferreira, Antonio J. Piantino Ferreira

**Affiliations:** 1 Department of Pathology, School of Veterinary Medicine, University of São Paulo, Av. Prof. Orlando Marques de Paiva, 87, São Paulo 05508-900, Brazil; ruychaconv@usp.br (R.D.C.); andrea.diazf@usp.br (A.J.D.F.); mbrito@usp.br (M.B.G.); csastolfi@gmail.com (C.S.A.-F.); 2 Department of Genetics, Physiology, and Microbiology, Faculty of Biology, Complutense University of Madrid (UCM), 28040 Madrid, Spain; chrsan01@ucm.es; 3 Faculty of Biological Sciences, National University of Trujillo, Trujillo 13001, La Libertad, Peru; pajuelosarah@gmail.com

**Keywords:** *Orthobornavirus*, Proventricular Dilatation Disease, nucleoprotein, matrix, selective pressure, phylodynamics, phylogeography

## Abstract

**Simple Summary:**

Parrot bornavirus (PaBV) causes Proventricular Dilatation Disease (PDD), a fatal neurological disorder in *Psittaciformes*. Its diversity in South America is poorly known. We detected the first case of a genotype 2 (PaBV-2) infection in *Cacatua galerita* in Brazil, which presented severe neuropathies and PDD. Evolutionary analysis estimated a potential Brazilian (or South American) ancestor as the origin of this genotype and possibly other genotypes of the *Orthobornavirus alphapsittaciforme* species. Additionally, a progressive decline in the size of the effective population was observed, which could be related to various factors, such as biodiversity loss and ecosystem disruption. The emergence or reemergence of PaBVs related to ancestral strains in Brazil and South America is a matter of concern, as the spread of these viruses poses a risk to bird biodiversity and endangered species. These results underscore the need for surveillance studies in this region.

**Abstract:**

Parrot bornavirus (PaBV) is an RNA virus that causes Proventricular Dilatation Disease (PDD), neurological disorders, and death in *Psittaciformes*. Its diversity in South America is poorly known. We examined a *Cacatua galerita* presenting neuropathies, PDD, and oculopathies as the main signs. We detected PaBV through reverse transcription polymerase chain reaction (RT-PCR) and partial sequencing of the nucleoprotein (*N*) and matrix (*M*) genes. Maximum likelihood and Bayesian phylogenetic inferences classified it as PaBV-2. The nucleotide identity of the sequenced strain ranged from 88.3% to 90.3% against genotype PaBV-2 and from 80.2% to 84.4% against other genotypes. Selective pressure analysis detected signs of episodic diversifying selection in both the *N* and *M* genes. No recombination events were detected. Phylodynamic analysis estimated the time to the most recent common ancestor (TMRCA) as the year 1758 for genotype PaBV-2 and the year 1049 for the *Orthobornavirus alphapsittaciforme* species. Substitution rates were estimated at 2.73 × 10^−4^ and 4.08 × 10^−4^ substitutions per year per site for *N* and *M*, respectively. The analysis of population dynamics showed a progressive decline in the effective population size during the last century. Timescale phylogeographic analysis revealed a potential South American ancestor as the origin of genotypes 1, 2, and 8. These results contribute to our knowledge of the evolutionary origin, diversity, and dynamics of PaBVs in South America and the world. Additionally, it highlights the importance of further studies in captive *Psittaciformes* and the potential impact on endangered wild birds.

## 1. Introduction

Parrot bornaviruses (PaBVs) are enveloped, non-segmented, single-stranded, negative-sense RNA viruses. They belong to the order *Mononegavirales*, the *Bornaviridae* family, and the *Orthobornavirus* genus [1]. This genus comprises two viral species that infect psittacines: *Orthobornavirus alphapsittaciforme* and *Orthobornavirus betapsittaciforme* [1,2].

Proventricular Dilatation Disease (PDD) is a progressively lethal neurological disease in psittacines [3]. PDD was first described in the 1970s in birds imported from Santa Cruz, Bolivia, and exhibited high lethality among macaws and other large psittacines [4]. The etiology of PDD remained unknown until 2008, when the detection of a parrot bornavirus (formerly avian bornavirus—ABV) was achieved [5,6].

To date, eight parrot bornaviruses (PaBVs) have been discovered in psittacine birds, belonging to the viral species *Orthobornavirus alphapsittaciforme* (PaBV-1 to PaBV-4, PaBV-7, and PaBV-8) and *Orthobornavirus betapsittaciforme* (PaBV-5 and PaBV-6) [2]. Genotypes PaBV-2 and PaBV-4 are the predominant ones in these birds [2]. PDD has been reported with prevalence in *Psittaciformes*. However, it has also been reported in more than 70 species of captive and free-ranging birds, including non-psittacine species belonging to the orders *Passeriformes*, *Anseriformes*, *Accipitriformes*, *Charadriiformes*, *Falconiformes*, *Pelecaniformes*, and *Piciformes* worldwide [7,8].

Infections caused by PaBVs affect the autonomic nerves of the digestive tract, leading to gastrointestinal dysfunction (dysphagia, regurgitation, and the passage of undigested food in feces) [3,8]. PaBVs are also associated with significant central nervous system damage, which may result in the development of encephalitis and myelitis, leading to depression, seizures, ataxia, blindness, and tremors [7]. Affected birds may exhibit both neurological and gastrointestinal signs [3,8,9]. However, PaBVs have also been detected in clinically healthy birds [10]. The prognosis for PDD-affected birds is poor, and no specific treatment is available to date. Birds can survive for months to years with symptomatic treatment [10].

In Brazil, known for its highly diverse avifauna and being the richest in psittacines, PaBVs have been detected in captive and free-ranging psittacines and *Passeriformes* [11,12,13]. These infections have mainly corresponded to the PaBV-4 genotype [11,12,14], with the PaBV-8 genotype unique to this country [15]. This study describes a severe case of PDD in a sulphur-crested cockatoo (Cacatua galerita) and includes comparative molecular and evolutionary analysis with other PaBV strains worldwide.

## 2. Materials and Methods

### 2.1. Clinical Case—History

A 15-year-old male sulphur-crested cockatoo weighing 0.37 kg was referred to the University of Sao Paulo Veterinary Teaching Hospital (HOVET-FMVZ/USP; São Paulo, SP, Brazil) with a 4-month history of depression and loss of appetite. The bird was kept in a garden with free-roaming access and shared its environment with two Amazon parrots. Additionally, the bird cohabited with two macaws in a separate aviary. The animal was acquired from a pet store in São Paulo State and had lived with the owner since it was a chick, until it reached approximately 13 years of age. After being raised by the family, the owners donated the bird to its current caregiver. The caregiver reported that it began exhibiting mild apathy symptoms shortly after taking possession of the bird. At the outset of the consultation, the patient’s owner expressed significant concerns about ocular symptoms, specifically swollen eyes. According to the caregiver, the bird had been manifesting these symptoms for an estimated 2-month period before the consultation.

A physical examination was performed, and the bird was noted to be alert and responsive but appeared weak and had difficulty maintaining balance. The patient’s feathers were unkempt, and the area around its eyes appeared inflamed. The veterinarian observed rotational nystagmus, a head tilt to the right, and a lack of coordination and ataxia. The referring veterinarian had previously observed these neurological signs and collected fecal samples to test for avian bornavirus. Due to the owner’s significant concern regarding the appearance of ocular symptoms, an ophthalmologic examination was conducted. The ophthalmologic exam revealed a discontinuity with loose epithelium in the left cornea and bilateral lens opacity. A fluorescein test was performed, which showed a positive result in the left eye. The bird was diagnosed with an indolent ulcer in the left eye, keratoconjunctivitis, and a cataract in the right eye. The treatment plan included 3 mg/mL tobramycin ophthalmic solution administered every 12 h in the left eye, 10% acetylcysteine ophthalmic solution every 12 h in the left eye, and Systane^®^ UL Lubricant Ophthalmic Solution (Systane, Macquarie Park, NSW, Australia) every 12 h in the right eye. The patient’s progress was regularly monitored through follow-up appointments to evaluate the response to treatment and adjust the plan as necessary. The ophthalmic condition showed improvement; however, despite this progress, the patient’s overall condition progressively worsened, with the development of new symptoms such as tremors and falling.

Despite the severity of the bird’s condition and the veterinarian’s explanations of the difficulties of treating the disease, the caregiver elected to treat the bird with homeopathy. The bird’s condition was treated with homeopathy for around eight months, during which periods of improvement and deterioration occurred. However, the bird’s condition declined over time, leading to progressive thinning, weakness, and decreased responsiveness to handling. Recognizing the poor prognosis and lack of response to therapy, the owner chose to euthanize the bird.

The study was conducted according to the guidelines of the Declaration of Helsinki and approved by the Ethics Commission on Animal Use of the School of Veterinary Medicine, University of São Paulo (FMVZUSP), under CEUAVET protocol no. 1517240614. In addition, written consent was obtained from the bird owner to develop this study.

### 2.2. Molecular Detection

A necropsy was performed on the euthanized bird. Samples of the brain, proventriculus, liver, spleen, and kidney were collected for molecular detection. The selected organs were macerated in sterile phosphate-buffered saline (PBS) at pH 7.0 in a 1:1 volume ratio. Then, centrifugation was performed at 12,000× *g* at 4 °C for 20 min, and 200 µL of supernatant was collected for the next step.

Nucleic acid extraction was performed with the BioGene DNA/RNA Viral Kit (Quibasa Química Básica Ltda., Belo Horizonte, MG, Brazil) according to the manufacturer’s recommendations. The viral RNA concentration was evaluated using a NanoDrop 2000 (Thermo Fisher Scientific, Wilmington, DE, USA). The detection of avian bornavirus was performed with RT-PCR assays targeting the nucleoprotein (*N*) and matrix (*M*) genes, following the primers and PCR conditions previously published [16]. These assays amplify products of approximately 389 and 352 bp in length. The PCR products of the sample with higher quantities visualized in the electrophoresis gel were purified using the Illustra^TM^ GFX PCR and Gel Band Purification Kit (GE Healthcare Biosciences, Piscataway, NJ, USA), following the manufacturer’s instructions. Sequencing was performed on a 3500xL Genetic Analyzer with the BigDye^TM^ Terminator v3.1 Cycle Sequencing Kit (Applied Biosystems, Carlsbad, CA, USA). Sequence reads were assembled using Geneious Prime^®^ 2020.2.4 and were deposited in GenBank under the accession numbers MN257630 (gene *N*) and MN257629 (gene *M*). The detected strain associated with the reported case was subsequently identified as USP-1295.

### 2.3. Sequence and Phylogenetic Analysis

Phylogenetic analysis was conducted on 130 sequences, comprising 128 *Orthobornavirus alphapsittaciformes* strains retrieved from GenBank, including the strain from this study, as well as two sequences belonging to the species *Orthobornavirus betapsittaciformes* (parrot bornavirus 5, PaBV-5) and the species *Orthobornavirus estrildidae* (estrildid finch bornavirus 1, EsBV-1) as outgroups [2]. The selection criteria included the availability of year and country information, as well as the availability of the genes *N* (nucleoprotein) and *M* (matrix), comprising the regions of interest (342 bp in gene *N* and 306 bp in gene *M*). Sequences were aligned using the MAFFT online service [17], edited, and concatenated with Geneious Prime v2020.2.4. The best-fit substitution models for phylogenetic analysis were estimated with ModelTest-NG v0.1.7 [18] using the Akaike information criterion (AIC). A maximum likelihood (ML) tree was constructed with RaxML-NG v1.1.0 [19], and nodal supports were estimated with 1000 bootstrap replicates to infer the phylogenetic relationships among the sequences. The phylogenetic tree was visualized with iTOL v6 [20]. The identity analysis was performed using SDT v1.2 [21].

### 2.4. Selective Pressure and Recombination Analysis

Gene-wide selective pressure was detected using the BUSTED method on the 128 sequences belonging to the viral species *Orthobornavirus alphapsittaciforme*. The SLAC, FEL, and FUBAR methods were also employed to detect sites experiencing pervasive diversifying or purifying selection. Finally, MEME was used to identify sites undergoing episodic diversifying selection. These methods were performed on the adaptive evolution server Datamonkey 2.0 [22].

A recombination analysis was conducted on the same 128 concatenated sequences of the *N* and *M* genes from PaBVs belonging to the viral species *Orthobornavirus alphapsittaciforme*, utilizing the RDP, Chimaera, BootScan, 3Seq, GENECONV, MaxChi, and SiScan methods implemented in the software RDP5 (v5.53) [23].

### 2.5. Phylodynamics and Phylogeography

The demographic history, evolutionary rates, and time-scaled phylogenetic tree of the concatenated *N* and *M* partial genes of the analyzed sequences were inferred using BEAST v2.6.7 [24]. The substitution model was selected using the BEAST Model Test, employing the relaxed log normal clock model with a coalescent constant population, using a Markov Chain Monte Carlo (MCMC) chain of 200 million generations and sampling every 1000 generations. The maximum clade credibility tree (MCC) was generated with a 20% burn-in using the TreeAnnotator software (v1.8) included in the BEAST package. The results were analyzed and visualized using Tracer v1.7.2 [25], and the time-scaled tree (tMRCA tree) was visualized using Figtree v1.4.4 [26].

To propose possible PaBV genotype geographical routes, the ancestral reconstruction and identification of the time to the most recent common ancestor (tMRCA) were performed using Nextstrain v4.2.0 [27]. The pipeline was built using Snakemake v7.14.2 [28]; augur v18.0 [29] was used to track the evolution from nucleotide alignment, using MAFFT v7.508 [17], iq-tree v1.6.12 [30], and TreeTime v0.9.4 [31] for the maximum likelihood phylogeny and timed tree inference, respectively. The results were then visualized using Auspice v2.38.0 [27].

## 3. Results

### 3.1. Clinical Case, Necropsy, and Molecular Detection

At necropsy, the left eyeball exhibited a slight protrusion. The lungs displayed a subtle reddish hue, while the heart showcased a mildly thickened left ventricular myocardium, accompanied by a reduction in ventricular cavity size. The ingluvies, proventriculus, and gastric ventricle contained solid matter intermingled with yellowish-white mucus, whereas the small intestine and colon exhibited moderate brownish content. The meninges within the central nervous system showed thickening and a whitish appearance with a gelatinous texture. No significant macroscopic alterations were evident in the remaining organs, including the tongue, thyroid, trachea, esophagus, spleen, liver, pancreas, kidneys, and adrenal glands.

After PCR analysis, avian bornavirus was detected in the brain, spleen, and liver samples. The liver sample showed a higher concentration of the amplified products and was chosen for sequencing.

### 3.2. Sequence and Phylogenetic Analysis

Sequences were based on the analyzed sequences’ concatenated *N* and *M* partial genes (648 bp in length). The analysis of sequence pairwise identity for the Brazilian strain USP-1295 showed higher values against the PaBV-2 sequences (88.3% to 90.3%) (Figure S1). Concerning the other genotypes of the viral species *Orthobornavirus alphapsittaciforme*, the values were lower (80.2% to 84.4%).

**Figure 1:**
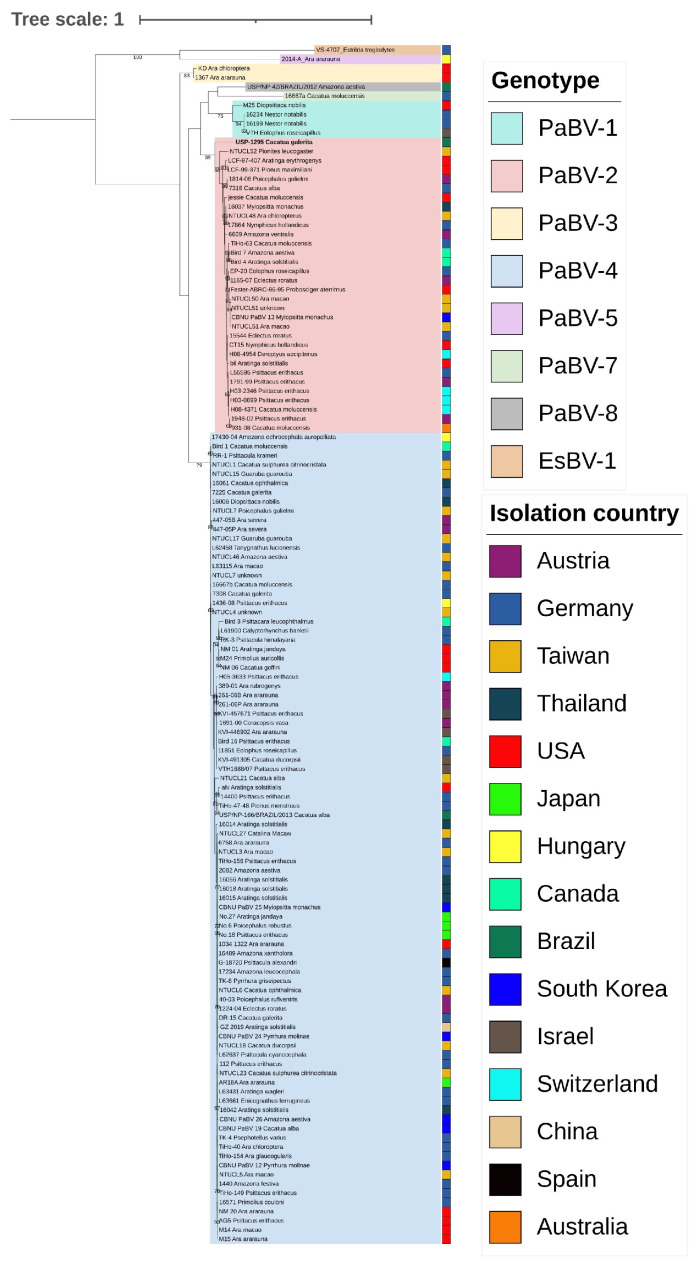
Maximum likelihood phylogenetic tree of the 130 studied sequences. Sequences consisted of concatenated *N* plus *M* partial genes (648 base pairs in length). The tree was inferred under an HKY+G model with support values based on 1000 bootstrap replicates indicated on nodes. Clusters for each genotype are shaded in different colors. The Brazilian strain from this study is highlighted in bold.

**Figure 2:**
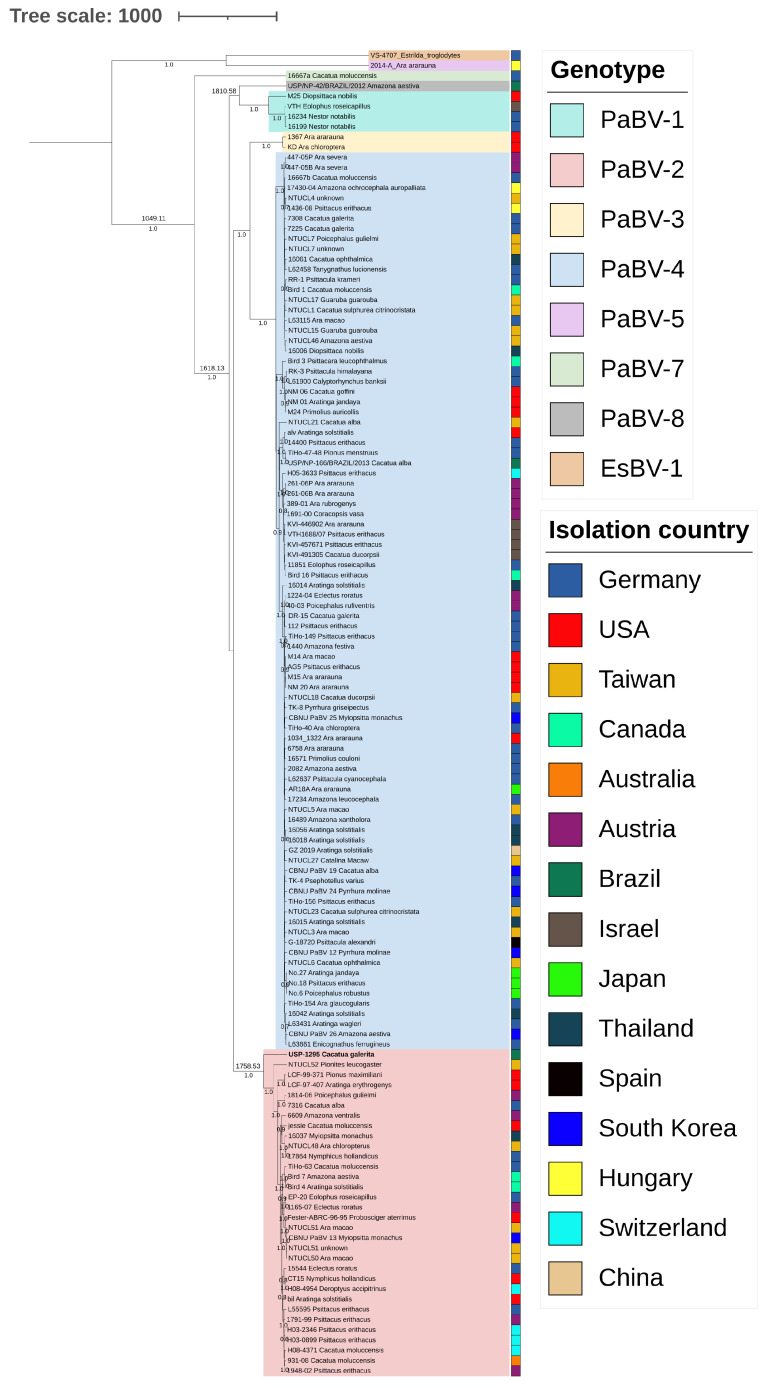
Posterior probability densities for the time to the most recent common ancestor (tMRCA) inferences for the 130 studied sequences. Sequences consisted of concatenated *N* plus *M* partial genes (648 base pairs in length). Posterior probabilities are indicated in the middle of each branch. Clusters for each genotype are shaded in different colors. The Brazilian strain from this study is highlighted in bold.

A phylogenetic tree, inferred using an ML method, was constructed using the substitution model HKY+G (Figure 1). This tree grouped the six genotypes of species *Orthobornavirus alphapsittaciforme* and separated them from the outgroup species. The Brazilian strain USP-1295 was grouped into genotype PaBV-2. Other Brazilian strains were clustered into genotypes 4 (USP/NP-166/BRAZIL/2013) and 8 (USP/NP-42/BRAZIL/2012). Similarly, the Bayesian concatenated tree differentiated the six genotypes of the species *Orthobornavirus alphapsittaciforme* and the Brazilian strain USP-1295 well, grouped again into genotype PaBV-2 (Figure 2).

Additionally, Bayesian trees inferred for individual partial gene *N* under the models TIM+G+I, K81+I, and TN93+G+I for codon positions +1, +2, and +3, respectively (Figure S2), and partial gene *M* under the models TN93+G, K81+G+I, and TN93+G+I for codon positions +1, +2, and +3, respectively (Figure S3), also clustered the Brazilian strain USP-1295 into genotype PaBV-2.

Selective pressure analysis identified eight sites under episodic diversifying (positive) selection in the analyzed fragments of the *N* and *M* genes (Table 1). At least one method determined and distributed these sites in the nucleoprotein (sites 255, 270, and 271) and matrix proteins (sites 26, 50, 58, 72, 94). Amino acid changes were also identified in some strains at each site under episodic positive selection. However, this did not occur with strain USP-1295 (or with the other Brazilian strains in the analysis), except for site 270, which presented the T270V amino acid change in two Brazilian strains and several other strains from different countries (Table 1).

No potential recombination events were detected in the evaluated sequences.

### 3.3. Phylodynamics and Phylogeography

The N and M concatenated sequences were partitioned: the *N* gene spanned positions 1 to 342, while the *M* gene spanned positions 343 to 648. The codon positions were partitioned as +1, +2, and +3. The BEAST Model Test was employed to determine the best models. For codon positions +1, +2, and +3 in partition *M*, the selected models were TN93+G, TIM+G+I, and TN93+G+I, respectively. In partition *N*, the models TIM+G+I, K81+I, and TN93+G+I were selected for codon positions +1, +2, and +3, respectively. Subsequently, the BEAST2 program estimated the time to the most recent common ancestor (tMRCA) for the Brazilian strain USP-1295 in 1758, with a 95% highest posterior density interval of 1682–1834 (Figure 2).

**Figure 3:**
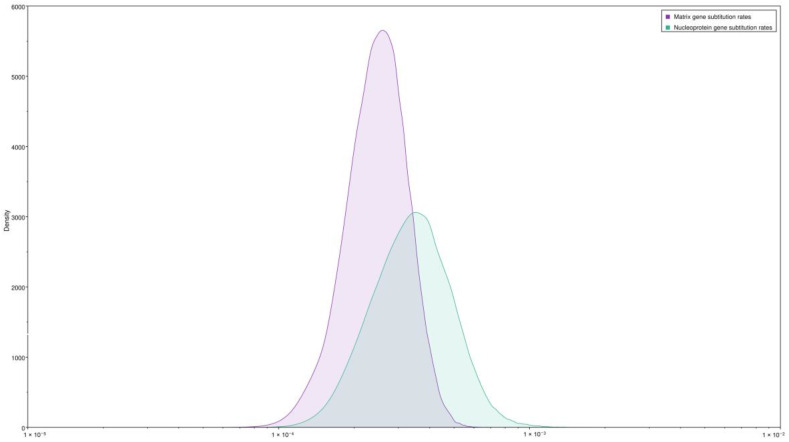
Mean evolutionary rates of the nucleoprotein and matrix partial genes for 128 parrot bornavirus strains belonging to the species *Orthobornavirus alphapsittaciforme* are represented as the number of substitutions per site per year.

In addition, we estimated the mean evolutionary rate for the *N* and *M* partial genes in the sequences belonging to the species *Orthobornavirus alphapsittaciforme*. The *N* gene exhibited a mean evolutionary rate of 2.73 × 10^−4^ substitutions per year per site, with a 95% highest posterior density interval of 1.39 × 10^−4^ to 4.15 × 10^−4^, and, for the *M* gene, the estimated mean evolutionary rate was 4.08 × 10^−4^ substitutions per year per site, with a 95% highest posterior density interval of 1.62 × 10^−4^ to 6.94 × 10^−4^ (Figure 3).

**Figure 4:**
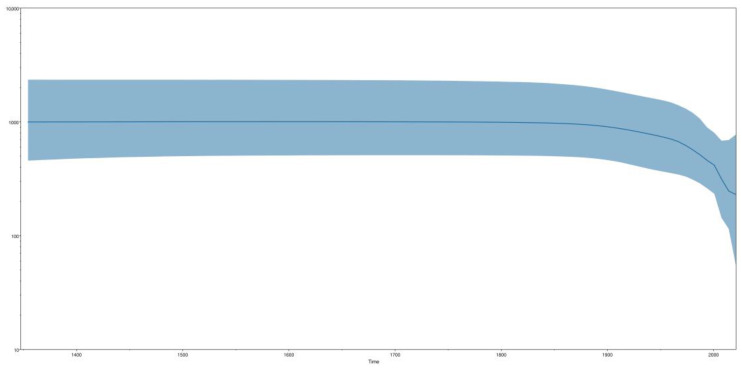
Bayesian skyline plot of 128 parrot bornavirus strains belonging to *Orthobornavirus alphapsittaciforme*. Sequences consisted of concatenated *N* plus *M* partial genes (648 base pairs in length). The *y*-axis represents the logarithm of the effective population size (Ne), and the *x*-axis represents the year. The center black lines represent the mean of Ne, and the shaded area represents Ne’s 95% highest posterior density (HPD).

Additionally, we constructed a Bayesian skyline plot (BSP) using the concatenated *N* and *M* partial genes in the sequences belonging to the species *Orthobornavirus alphapsittaciforme*. The BSP analysis (Figure 4) provided insights into the population dynamics across two distinct periods. The first period showcased a prolonged phase of stability, characterized by a relatively constant effective population size spanning from the mid-1450s to approximately the 1900s. However, the second period indicated a sharp decline in the effective population size, persisting up to the present time. 

**Figure 5:**
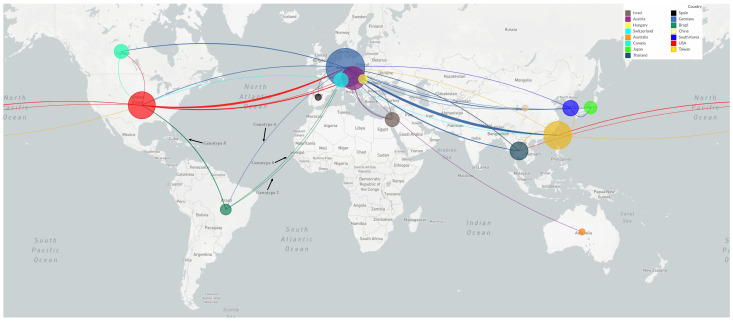
Global phylogeographic distribution of 128 parrot bornavirus strains belonging to *Orthobornavirus alphapsittaciforme*. Sequences consisted of concatenated *N* plus *M* partial genes (648 base pairs in length). Colored circles are proportional to the number of available sequences. Connecting lines are colored by the country of origin for each transmission route. The arrows indicate the direction of the spread of the Brazilian genotypes.

The phylogeographic analysis conducted by Nextstrain indicates that the transmission of genotype 8 of PaBV could have originated in Brazil around the 1320s (Figure 5). Specifically, it started with an ancestor of the strain USP/NP-42 Brazil 2012. It then spread to the United States by the 1800s and Germany by the 1930s and continued from Germany to Israel (Figure S4). Furthermore, the spread of genotype 2 began with an ancestor of the strain USP-1295 in Brazil around the 1500s (Figure 5). It was then transmitted to Switzerland. Subsequently, it spread through two routes: one towards the United States and Taiwan, respectively, around the 1700s, and later from the United States to Europe and East Asia around the 1820s (Figure S5). Similarly, the appearance of genotype 4 in Brazil could be a case of importation, as the strain USP/NP-166 Brazil 2013 might have originated in Germany (Figure 5 and Figure S6).

## 4. Discussion

Parrot bornaviruses can infect a wide diversity of captive and free-living birds, including vulnerable and endangered species [7,8]. Brazil is the country with the third-highest total diversity of birds (n = 1816) and includes many Important Areas for Birds and Biodiversity (IBAs) (n = 233). However, it is also the country with the second-highest number of globally threatened birds (n = 153), including 17 species of parrots [32,33].

Our avian bornavirus case, consistent with prior research, exhibited significant neurological signs [3,8], including ocular involvement, at times reported as a clinical sign [34], supporting the established association of the disease with central nervous system damage. The source of infection remains unknown, but studies suggest that PaBV-4 could enter through the olfactory pathway, causing lethal encephalitis responsible for the observed neurological signs. The virus likely followed proposed pathways, involving centrifugal spread from the brain through the spinal cord, impacting the parasympathetic and sympathetic nerves, and affecting the innervated organs [8].

Studies involving phylogenetic inferences provide an effective and precise way to understand viral diversity in specific populations or globally. One strategy for viral phylogenetic analysis involves concatenating sequences from two or more genes, which enhances the phylogenetic signal compared to studies based on individual genes or entire genomes. This approach increases the statistical power of molecular evolution analysis and improves the accuracy of phylogenetic trees by providing a larger number of substitutions [35,36,37,38]. Several phylogenetic studies on avian bornaviruses have included partial *N* and *M* genes separately, as these are the most abundant available sequences for PaBVs [15,16,39,40,41]. This study incorporated a comprehensive set of PaBV sequences and available epidemiological information to present a global view of this virus’s diversity. In Brazil, a few molecular studies with phylogenetic analyses on avian bornaviruses have mainly indicated the presence of the PaBV-4 genotype in captive and free-living birds [11,12,14]. PaBV-8 was identified and proposed as a new genotype but has only been detected in Brazil [15]. In the present study, phylogenetic inferences consistently classified our studied strain as the PaBV-2 genotype. Therefore, to our knowledge, this is the first report of PaBV-2 in Brazil and South America.

Recombination is an important evolutionary mechanism that contributes to genetic diversity through the emergence of new strains and genotypes. However, there are no reports of these events in PaBVs, including the present study. Recombination has never convincingly been documented in species of the *Mononegavirales*, and it is speculated whether this reflects an inability of these viruses to recombine because of the formation of the ribonucleoprotein, which inhibits the copy choice of the RNA-dependent RNA polymerase [42,43,44].

The survival and adaptation of the virus in a specific environment are influenced by mechanisms of selective pressure, which can be either biotic or abiotic. Few studies have detected signs of selective pressure in *Orthobornaviruses* [45,46]. In our study, we observed the presence of sites undergoing episodic diversifying selection in both the *N* and *M* genes. Interestingly, diversifying (positive) selection events have been previously reported in the nucleoprotein of *Orthobornaviruses* and have been associated with host adaptation [45], as well as in other *Mononegavirales* such as ebolaviruses [47]. Therefore, the presence of these sites in the nucleoprotein and the matrix may underscore their significance in the diversification and adaptation of PaBVs to novel hosts and environments.

The evolutionary rates of nucleotide substitution in the analyzed genotypes belonging to *Orthobornavirus alphapsittaciforme* showed similar ranges between the nucleoprotein and the matrix, aligning with the evolutionary rates of other *Mononegavirales* [48,49], including other bornaviruses [46,50]. It was estimated that the time to the most recent common ancestor (TMRCA) of the entire species dates to the beginning of the 11th century, while genotypes 4 and 2, which are currently the most prevalent, would have originated in the mid-17th and mid-18th centuries, respectively. These results, indicating a temporally distant origin several centuries ago, align with findings by He et al. [50], who estimated the date of origin of avian bornaviruses to be around the 13th century. Interestingly, the discovery and analysis of the ancestry of endogenous bornavirus-like elements (EBLs) in various vertebrates, including humans, have indicated an ancient age of millions of years for bornaviruses [44,51,52]. Notably, in the case of PaBV-2, the Brazilian strain USP-1295 (present study) reflects a basal divergence within the genotype, suggesting a common ancestor from Brazil or South America. This result aligns with the basal divergence shown by the USP/NP-42 strain, representative of the genotype PaBV-8, which has been exclusively detected in Brazil. Therefore, hypotheses emerge, situating Brazil (or South America) as a potential ancestral source and reservoir of PaBVs or a source of divergent representative species. These hypotheses are in accordance with the first reported event describing PDD signs in birds imported from Bolivia [4]. Interestingly, other studies have also identified basal clades or branches from divergent viruses detected in Brazil [53,54], raising concerns when unexplored ecosystems that may contain these and other viral species are contacted, altered, or destroyed.

Our results show a noticeable and accentuating decrease in the effective population size of PaBVs in recent decades. This loss of viral diversity could be an indicator of the growing number of birds in vulnerable and endangered situations, as well as the increasing loss of ecosystems and the effects of climate change worldwide [55]. The genomic diversity of viruses results from continuous and dynamic ecological and evolutionary processes influenced by the host [56,57]. Smaller effective population sizes are associated with a more robust genetic drift process, and a reduction in biodiversity can facilitate pathogen transmission by increasing the number of competent hosts [56,58]. The detection of an ancestral-descendent PaBV-2 strain in an old-world Cacatua is supported by the hypothesis of native strains of PaBVs being transmitted from the new world to a competent host. Deforestation and human land use altering the natural high-diversity habitats result in the large-scale loss and fragmentation of habitats and wildlife populations of both hosts and pathogens [59,60,61].

The illegal wildlife trade facilitates the transmission of viruses [62]. Birds are subjected to harsh conditions during capture and transport, resulting in many fatalities, or may become more susceptible to disease due to stress and immunosuppression [63]. This situation is prevalent in Brazil, where studies have identified pathogenic viruses, including bornaviruses, in rescued or confiscated birds from the illegal wildlife trade [11,12,13,15]. This issue is not limited to *Psittaciformes*, as Brazil ranks among the top countries engaged in the illegal trade of wildlife birds. As a result, between 1990 and 2020, up to 32 pathogens, some of which have zoonotic potential, may have been imported [64].

Research has revealed ancient bornaviral infections across various vertebrate lineages spanning nearly 100 million years, indicating a longstanding coexistence between these viruses and their hosts [65]. Due to the severe neurological effects observed, it is imperative to continue surveillance studies on bornaviruses to monitor infections that pose a potential threat to endangered birds. Furthermore, since PaBVs can infect species beyond Passeriformes, they represent a risk of spreading to new species, environments, and different geographical zones based on the migration patterns and habitat preferences of these species [2].

Finally, it is essential to mention some limitations of the present study. Due to the limited availability of complete genomes of PaBVs worldwide, the approach used in the present study aimed to include all the homologous sequences available to date and analyze them with robust bioinformatics tools to approximate the current global scenario appropriately. However, these results can be improved as more genomes become available in the future.

## 5. Conclusions

In conclusion, this study identified the first case of PaBV-2 infection in Brazil and South America. While PaBV-2 is one of the most common genotypes, phylodynamic and phylogeographic analyses suggested a potential Brazilian (or South American) origin for this genotype and possibly other genotypes within the *Orthobornavirus alphapsittaciforme* species.

This study detected high substitution rates and episodic positive selection events in PaBVs. However, it also revealed a progressive decline in the effective population size, which could be attributed to various factors, including biodiversity loss and ecosystem disruption. The emergence of an ancestral-related PaBV-2 strain highlights the need for monitoring and control measures for this globally distributed virus. The spread of emerging or reemerging pathogens can significantly impact conservation efforts for threatened species.

## Figures and Tables

**Table 1:** Amino acid changes and sites under episodic diversifying (positive) selection pressure estimated by Datamonkey methods in the analyzed nucleoprotein and matrix partial proteins belonging to the species *Orthobornavirus alphapsittaciforme*.

	Selective Pressure																						
	MEME																						
	BUSTED (ALL)																						
	Protein	Nucleoprotein										Matrix											
Genotype	Position	216	255	265	269	270	271	272	273	276	280	26	32	34	46	50	58	65	72	77	84	94	98
	Strain	D	L	K	D	T	T	A	K	A	D	I	T	H	K	Q	V	V	T	F	V	K	K
PaBV-2	**USP-1295_Cacatua_galerita_Brazil_2019**	*	*	*	*	V	*	T	*	*	*	*	*	*	R	*	*	*	*	*	*	*	*
	1791-99_Psittacus_erithacus_Austria_1999	*	*	*	*	V	*	T	*	*	*	*	*	*	*	*	M	*	*	*	*	*	*
	H03-2346_Psittacus_erithacus_Switzerland_2003	*	*	*	*	V	*	T	*	*	*	*	*	*	*	*	*	*	*	L	*	*	*
	Fester-ABRC-96-95_Probosciger_aterrimus_USA_2006	*	*	*	*	V	*	T	*	*	*	*	*	*	R	R	*	*	*	*	*	*	*
	bil_Aratinga_solstitialis_USA_2006	*	*	*	*	V	*	T	*	*	*	V	*	*	*	*	*	*	*	*	*	*	*
	931-08_Cacatua_moluccensis_Australia_2008	*	*	*	*	V	*	T	*	*	*	*	*	*	*	E	*	*	*	*	*	*	*
	17864_Nymphicus_hollandicus_Germany_2011	*	*	*	*	V	*	T	*	*	*	*	I	*	R	*	*	*	*	*	*	*	*
	NTUCL52_Pionites_leucogaster_Taiwan_2017	*	*	*	*	I	*	T	*	*	*	*	*	*	R	*	*	*	*	*	*	*	*
PaBV-3	KD_Ara_chloroptera_USA_2007	*	*	*	N	I	*	T	T	*	N	*	*	*	R	*	*	I	*	L	I	*	*
	1367_Ara_ararauna_USA_2008	*	*	*	*	I	*	T	T	*	*	*	*	*	R	*	*	I	*	L	I	*	*
PaBV-4	40-03_Poicephalus_rufiventris_Austria_2003	*	*	*	*	M	*	*	*	*	*	*	*	*	*	*	*	*	*	L	*	*	*
	KVI-446902_Ara_ararauna_Israel_2004	*	*	*	*	*	*	*	*	*	*	*	A	*	R	*	*	*	*	*	*	*	*
	447-05B_Ara_severa_Austria_2005	*	*	*	*	*	I	*	*	*	*	*	*	*	*	*	*	*	*	*	*	*	R
	261-06P_Ara_ararauna_Austria_2006	*	V	*	*	*	*	*	*	*	*	*	*	*	R	*	*	*	*	*	*	*	*
	AG5_Psittacus_erithacus_USA_2008	*	*	*	*	*	*	*	*	*	*	*	*	*	*	*	G	*	*	*	*	*	R
	6758_Ara_ararauna_Germany_2008	*	*	*	*	*	*	*	*	*	*	*	*	*	*	*	*	*	*	*	*	*	*
	**USP/NP-166/BRAZIL/2013_Cacatua_alba_Brazil_2013**	*	*	*	*	*	*	*	*	*	*	*	*	*	*	*	*	*	*	*	*	*	*
	16042_Aratinga_solstitialis_Thailand_2016	*	*	*	*	*	*	*	*	*	*	*	*	R	*	*	*	*	*	*	*	R	*
	NTUCL7_Poicephalus_gulielmi_Taiwan_2016	*	*	*	*	*	A	*	*	*	*	*	*	*	*	*	*	*	*	*	*	*	*
	DR-15_Cacatua_galerita_Germany_2017	*	*	*	*	*	*	*	*	*	*	*	*	*	*	*	*	*	I	*	*	*	*
PaBV-1	VTH_Eolophus_roseicapillus_Israel_2006	*	*	*	E	I	*	T	*	T	*	*	*	*	*	*	*	*	*	*	*	E	*
	M25_Diopsittaca_nobilis_USA_2008	*	*	R	*	V	*	T	*	*	*	*	*	*	R	*	*	*	*	*	*	*	*
	16234_Nestor_notabilis_Germany_2011	*	*	*	E	I	*	*	*	T	*	*	*	*	*	*	*	*	*	*	*	*	*
PaBV-7	16667a_Cacatua_moluccensis_Germany_2010	*	*	R	E	*	*	*	*	T	E	*	*	R	*	*	*	*	L	*	*	*	*
PaBV-8	**USP/NP-42/BRAZIL/2012_Amazona_aestiva_Brazil_2012**	E	*	*	*	V	*	T	*	*	*	*	I	*	*	*	*	*	*	*	*	*	*

Amino acid substitutions: (*) = conserved (colored) polymorphism. Brazilian strains are highlighted in bold. BUSTED and MEME evidence ratio thresholds (*p* < 0.1).

## Data Availability

The obtained sequences were submitted to GenBank under the accession numbers MN257630 (gene *N*) and MN257629 (gene *M*).

## Supplementary Materials

The following supporting information can be downloaded at https://www.mdpi.com/article/10.3390/ani14010047/s1, Figure S1: Pairwise identity matrix of 128 parrot bornavirus strains belonging to Orthobornavirus alphapsittaciforme; Figure S2: Time-resolved maximum clade credibility tree (MCC) of the 130 studied sequences based on the N partial gene (342 base pairs in length); Figure S3: Time-resolved maximum clade credibility tree (MCC) of the 130 studied sequences based on the M partial gene (306 base pairs in length); Figure S4: Global phylogeographic distribution of parrot bornavirus genotype 8 strains (PaBV-8); Figure S5: Global phylogeographic distribution of parrot bornavirus genotype 2 strains (PaBV-2). Figure S6: Global phylogeographic distribution of parrot bornavirus genotype 4 strains (PaBV-4).
