# Supplementary material for: Evolutionary Analysis of a Parrot Bornavirus 2 Detected in a Sulphur-Crested Cockatoo (Cacatua galerita) Suggests a South American Ancestor
Source: Animals (Basel). 2023 Dec 22;14(1):47. doi: 10.3390/ani14010047 (PMC10778322; doi:10.3390/ani14010047)
Supplement: Supplementary file 1 [file animals-14-00047-s001.zip › animals-2701576-supplementary.pdf]

animals

Supplementary Materials

Evolutionary analysis of a parrot bornavirus 2 detected in a Sulphur-crested Cockatoo (*Cacatua galerita*) suggests a South American ancestor

Ruy D. Chacón [1,†], Christian J. Sánchez-Llatas [2,†], Andrea J. Diaz Forero [1], Marta B. Guimarães [1], Sarah L. Pajuelo [3], Claudete S. Astolfi-Ferreira [1] and Antonio J. Piantino Ferreira [1,*]

[1] Department of Pathology, School of Veterinary Medicine, University of São Paulo, Av. Prof. Orlando Marques de Paiva, 87, São Paulo 05508-900, Brazil; ruychaconv@usp.br (R.D.C.); andrea.diazf@usp.br (A.J.D.F.); mbrito@usp.br (M.B.G.); csastolfi@gmail.com (C.S.A.-F.)

2 Department of Genetics, Physiology, and Microbiology, Faculty of Biology, Complutense University of Madrid (UCM), 28040 Madrid, Spain; chrsan01@ucm.es (C.J.S.-L.)

3 Faculty of Biological Sciences, National University of Trujillo, Trujillo 13001, La Libertad, Peru; pajuelosarah@gmail.com (S.L.P.)

* Correspondence: ajpferr@usp.br (A.J.P.F.); Tel.: +55-11-3091-1352

† These authors contributed equally to this work

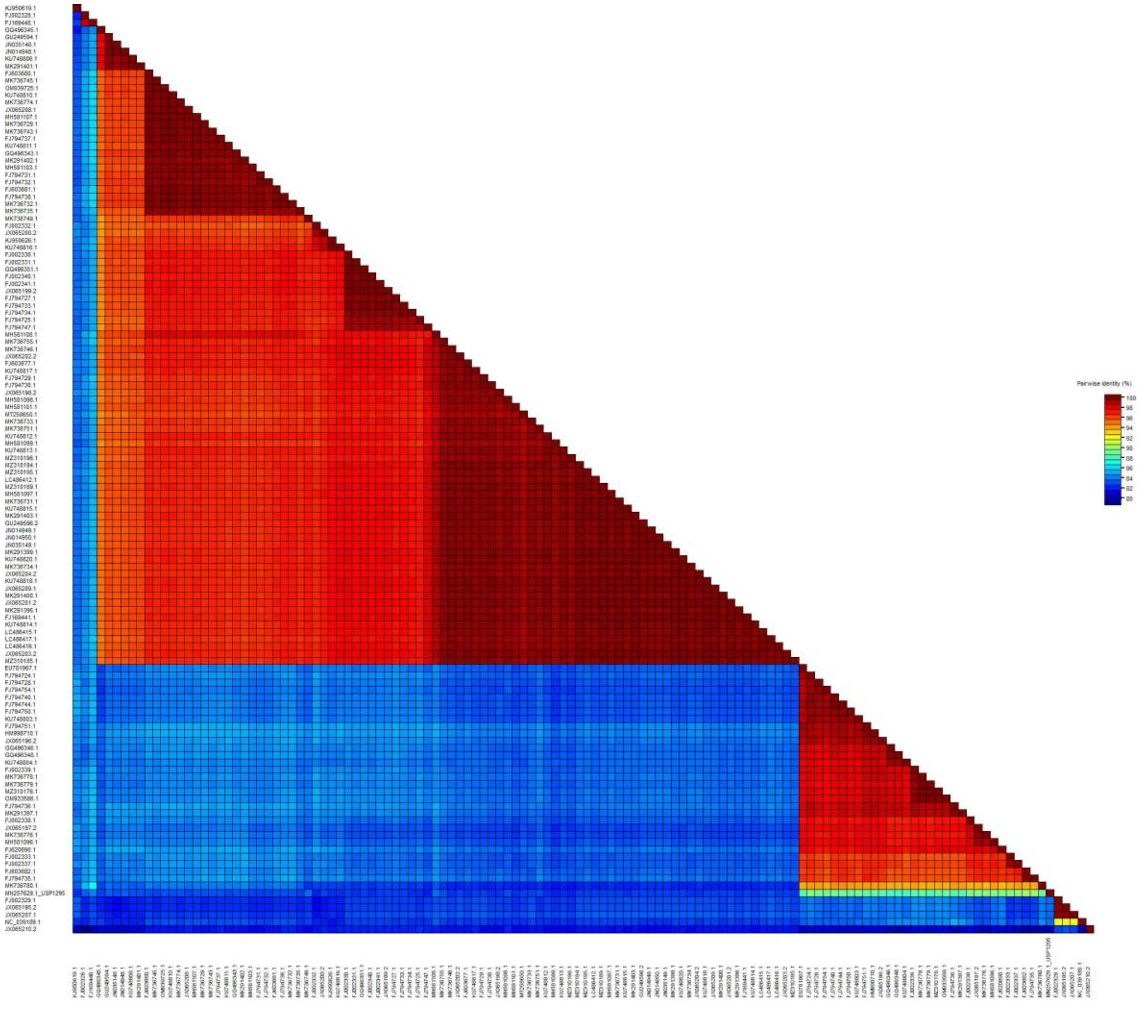

Figure S1. Pairwise identity matrix of 128 Parrot bornavirus strains belonging to *Orthobornavirus alphapsittaciforme*. Sequences consisted of concatenated N and M partial genes (648 base pairs in length). The Brazilian strain from this study is enclosed in a rectangle.

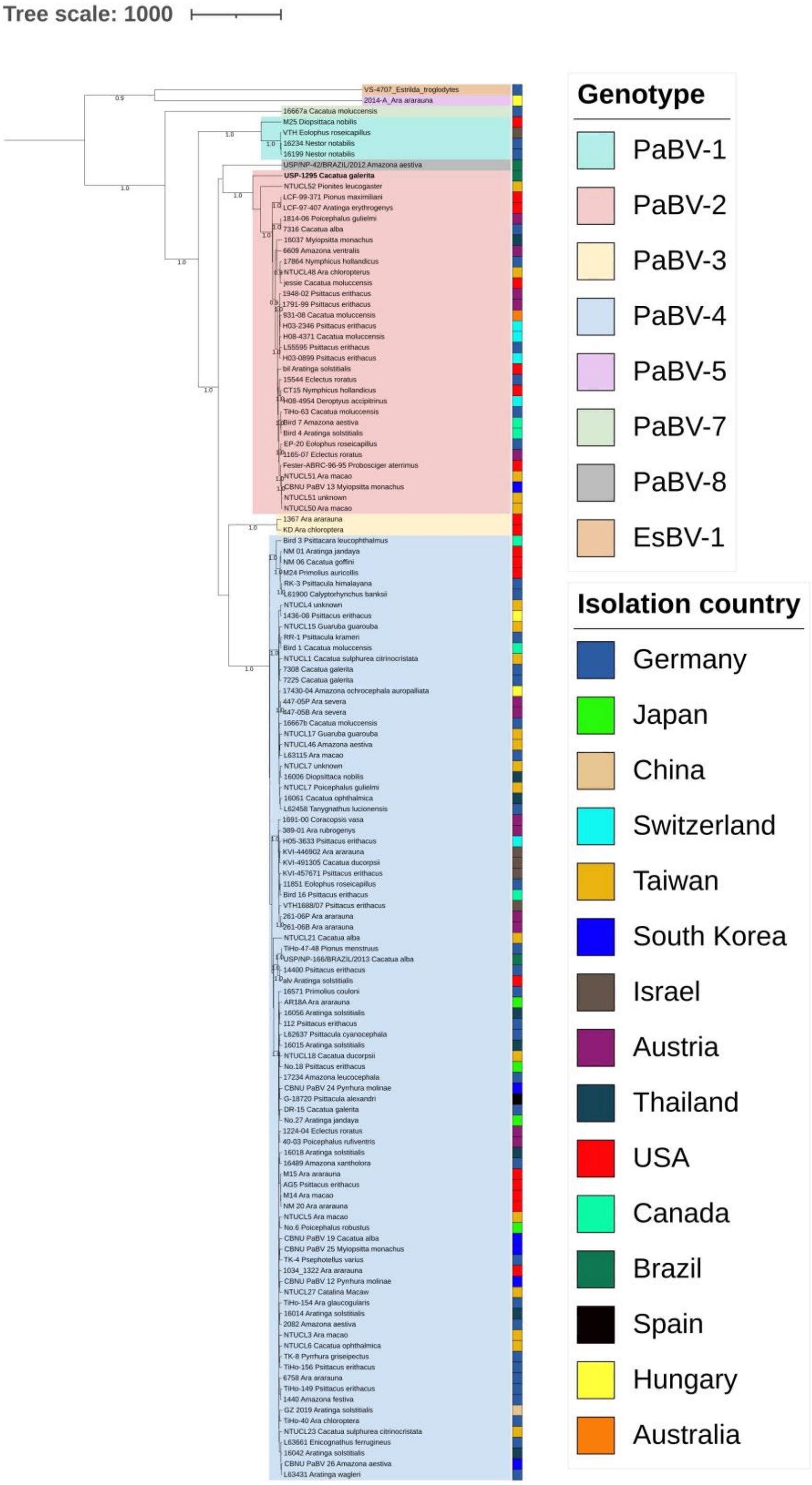

Figure S2. Time-resolved maximum clade credibility tree (MCC) of the 130 studied sequences based on the *N* partial gene (342 base pairs in length). The tree was inferred under the models TIM+G+I, K81+I, and TN93+G+I for codon positions +1, +2, and +3, respectively. Posterior probabilities are indicated in the middle of each branch, and clusters for each genotype are shaded in different colors. The Brazilian strain from this study is highlighted in bold.

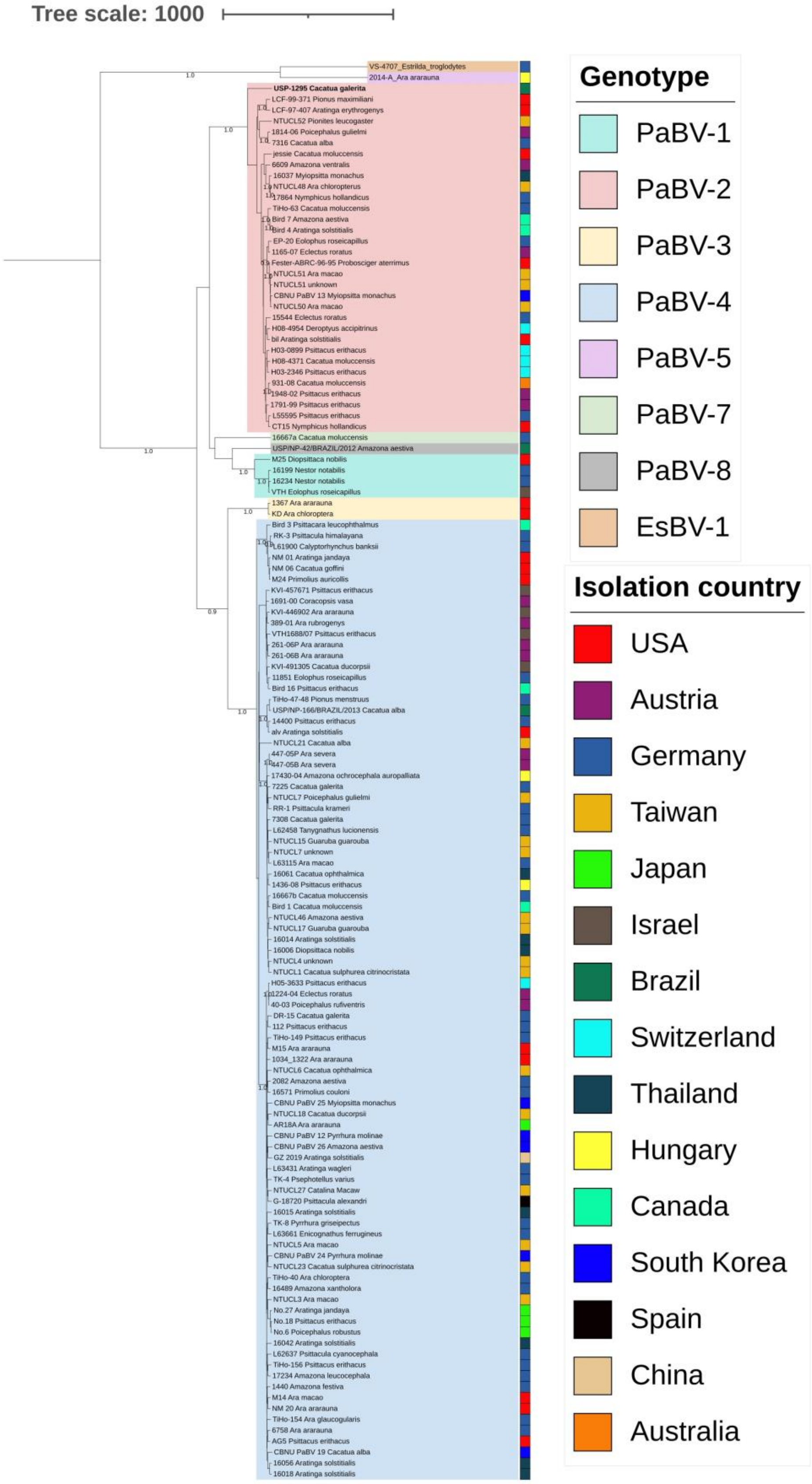

Figure S3. Time-resolved maximum clade credibility tree (MCC) of the 130 studied sequences based on the *M* partial gene (306 base pairs in length). The tree was inferred under the models TN93+G, K81+G+I, and TN93+G+I for codon positions +1, +2, and +3, respectively. Posterior probabilities are indicated in the middle of each branch, and clusters for each genotype are shaded in different colors. The Brazilian strain from this study is highlighted in bold.

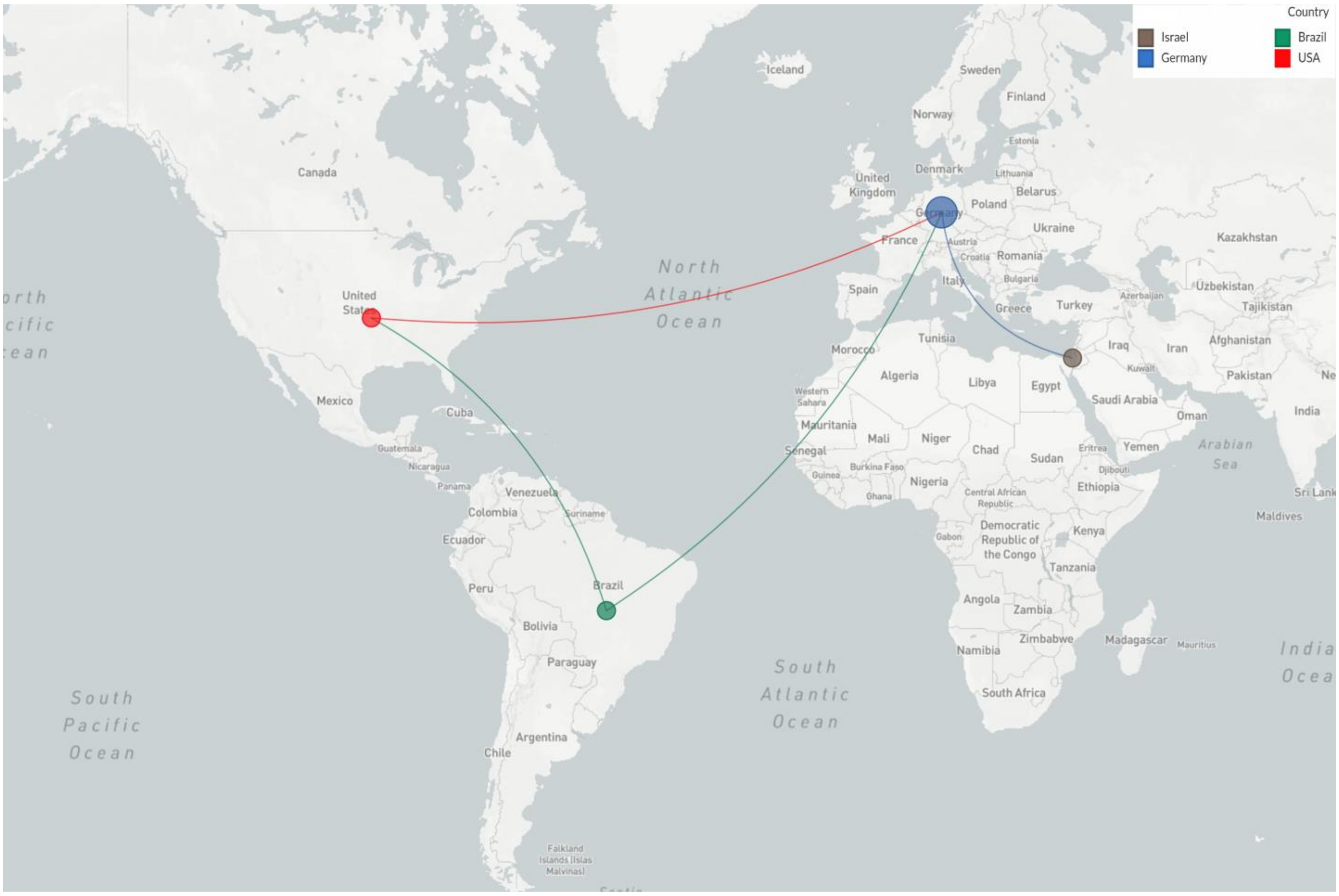

Figure S4. Global phylogeographic distribution of Parrot bornavirus Genotype 8 strains (PaBV-8). Sequences consist of concatenated *N* plus *M* partial genes (648 base pairs in length). Colored circles are proportional to the number of available sequences. Connecting lines are colored by the country of origin for each route of transmission. The arrows indicate the direction of the spread of the Brazilian genotypes.

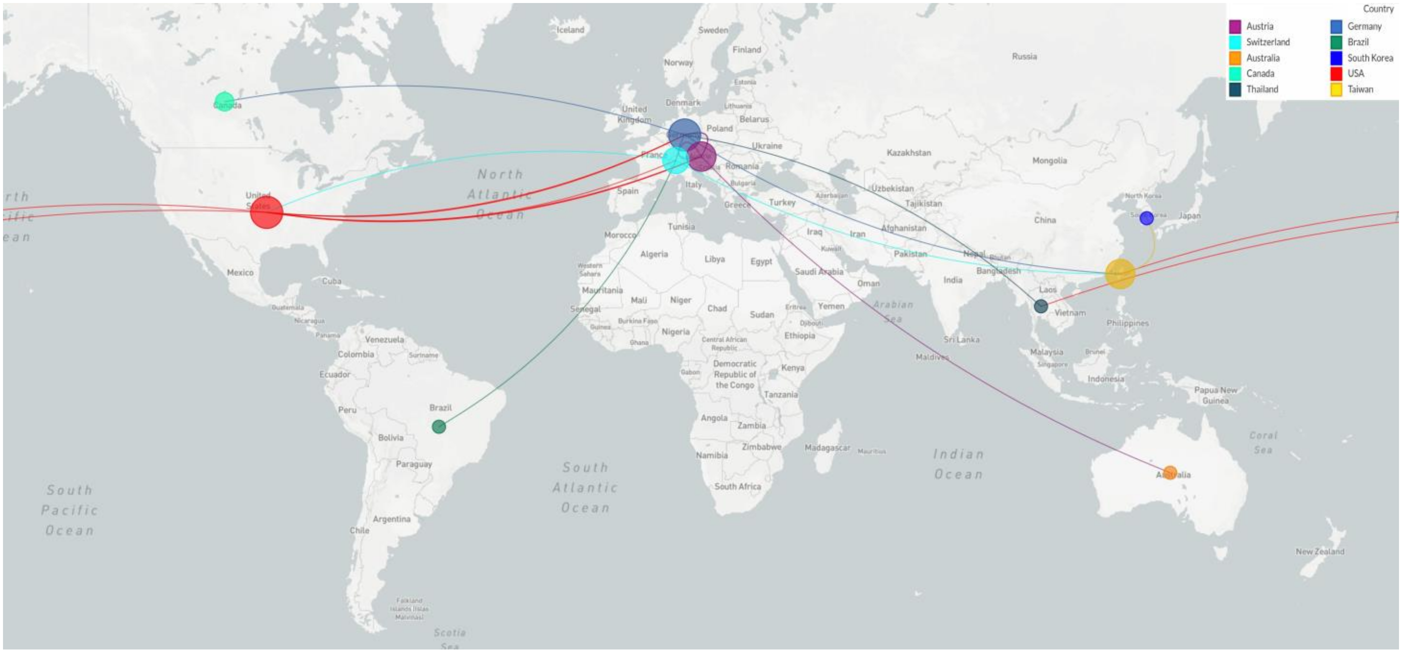

Figure S5. Global phylogeographic distribution of Parrot bornavirus Genotype 2 strains (PaBV-2). Sequences consist of concatenated *N* plus *M* partial genes (648 base pairs in length). Colored circles are proportional to the number of available sequences. Connecting lines are colored by the country of origin for each route of transmission. The arrows indicate the direction of the spread of the Brazilian genotypes.

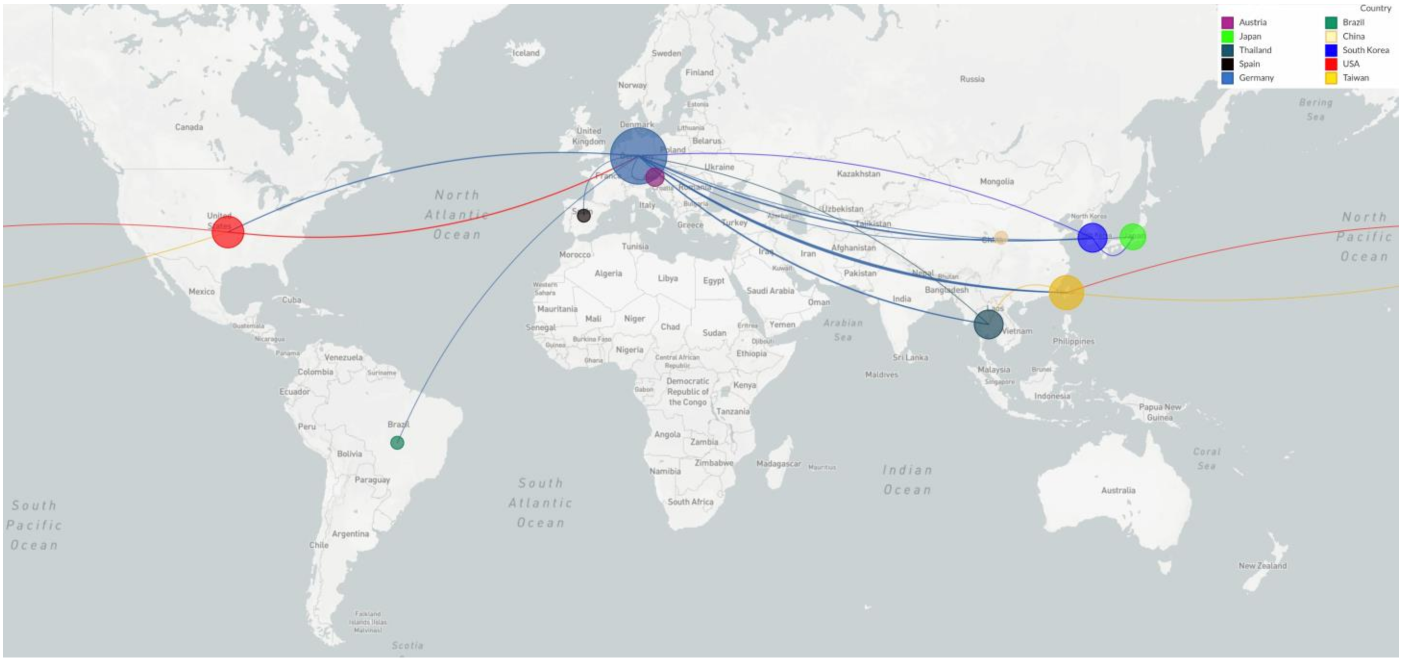

Figure S6. Global phylogeographic distribution of Parrot bornavirus Genotype 4 strains (PaBV-4). Sequences consist of concatenated *N* plus *M* partial genes (648 base pairs in length). Colored circles are proportional to the number of available sequences. Connecting lines are colored by the country of origin for each route of transmission. The arrows indicate the direction of the spread of the Brazilian genotypes.
